# Examining the Effects of Visibility and Time Headway on the Takeover Risk during Conditionally Automated Driving

**DOI:** 10.3390/ijerph192113904

**Published:** 2022-10-26

**Authors:** Haorong Peng, Feng Chen, Peiyan Chen

**Affiliations:** 1 Tongji Architectural Design (Group) Co., Ltd., 1230 Siping Road, Yangpu, Shanghai 200092, China; 2 Shanghai Research Center for Smart Mobility and Road Safety, Shanghai 200092, China; 3 The Key Laboratory of Road and Traffic Engineering, Ministry of Education, Tongji University, 4800 Cao’an Road, Jiading, Shanghai 201804, China

**Keywords:** conditionally automated driving, takeover performance, driving simulator, fog condition, time headway

## Abstract

The objective of this study is to examine the effects of visibility and time headway on the takeover performance in L3 automated driving. Both non-critical and critical driving scenarios were considered by changing the acceleration value of the leading vehicle. A driving simulator experiment with 18 driving scenarios was conducted and 30 participants complete the experiment. Based on the data obtained from the experiment, the takeover reaction time, takeover control time, and takeover responses were analyzed. The minimum Time-To-Collision (Min TTC) was used to measure the takeover risk level and a binary logit model for takeover risk levels was estimated. The results indicate that the visibility distance (VD) has no significant effects on the takeover control time, while the time headway (THW) and the acceleration of the leading vehicle (ALV) could affect the takeover control time significantly; most of the participants would push the gas pedal to accelerate the ego vehicle as the takeover response under non-critical scenarios, while braking was the dominant takeover response for participants in critical driving scenarios; decreasing the TCT and taking the appropriate takeover response would reduce the takeover risk significantly, so it is suggested that the automation system should provide the driver with the urgency of the situation ahead and the tips for takeover responses by audio prompts or the head-up display. This study is expected to facilitate the overall understanding of the effects of visibility and time headway on the takeover performance in conditionally automated driving.

## 1. Introduction

Automated driving is expected to improve road safety by reducing traffic accidents caused by human error [1]. In recent years, many companies and authorities have made great efforts to promote the development of automated vehicles [2]. According to the SAE standard, vehicle automation systems are categorized into 6 levels from L0 to L5 (full automation) [3]. Due to the limitations of technologies, law, and ethics issues, it is believed that realizing full automation on public roads still requires time, while conditional automation (L3) is expected to be widely applied in the near future [4]. At L3 driving automation, the vehicle is capable of conducting the entire dynamic driving tasks (DDTs) such as monitoring the environment and self-control under limited conditions. Drivers are not required to monitor the roadway and are able to perform non-driving-related tasks (NDRTs) within the operating design domain (ODD) of the L3 automated system [3]. When the vehicle is exiting its ODD or encounters an automation system failure, a takeover request (TOR) would be issued by the system, and the driver is required to take over the vehicle control within a limited time during the L3 automated driving to continue with the driving task or avoid any potential accidents [4]. Although the conditional automation system would bring about many benefits to drivers by allowing drivers to delegate driving tasks to the automated driving system, the safety issue during takeover is the most substantial concern which needs to be addressed when promoting L3 automation vehicles [5].

Various studies focusing on the takeover issue of L3 automation vehicles have been conducted during the past decade. Considering the potential risk of takeover in the on-road experiments, driving simulator experiments were applied to conduct the studies in most of the relevant literature [6]. According to the level of urgency of the takeover situation, driving scenarios used in these studies could be divided into two categories: critical and non-critical [7,8,9]. A critical scenario means that the TOR is issued in an unplanned manner and the driver is required to take over the vehicle control in a critical situation (such as an automation system malfunction caused by adverse weather or unexpected lane obstacle detection) [5]. A non-critical scenario means that the TOR takes place in a planned manner when the vehicle is about to leave the ODD areas, such as entering a ramp or rural roads without traffic signs and markings [9]. Most of the previous studies focused on critical takeover scenarios, often with the driving tasks of avoiding collisions ahead by braking or changing lanes [4,10,11]. Factors influencing the safety of takeover process in critical scenarios, such as driver characteristics [4], time-budget [11], takeover requests [12], non-driving related tasks [10], and road conditions [13], have been investigated in recent years, and meaningful findings and suggestions were obtained. For the non-critical scenarios, the literature on the takeover issue of L3 automation vehicles is limited [8]. Eriksson and Stanton [14] performed a takeover experiment for non-critical scenarios without time pressure, suggesting that designers of automated vehicles should take a wider range of takeover times into consideration rather than just focusing on the mean or median values. Pampel et al. [7] investigated the impact of unplanned and planned TOR on the takeover quality in non-critical situations by a simulator experiment. Xu et al. [8] investigated drivers’ takeover performance and workload under varying automation levels, time budgets, and road curvatures in driving scenarios wherein the ego vehicle was about to leave the highway. Since non-critical takeovers would be more common than critical takeovers, more studies focusing on the takeover safety issues in non-critical scenarios should be conducted to promote the development of L3 automation vehicles.

Car following is one of the most common situations that drivers encounter on the roads [15]. It is of great importance to investigate the safety issues relating to the takeover in the situation of car following. The car-following scenarios were used in some driving simulator experiments to measure drivers’ takeover performance in L3 automated driving [16,17]. In the experiment of Lu et al. [16], the car-following distance was set to 44.44 m by the system under different modes, representing a 2 s time headway (THW) to the leading vehicle. Two kinds of critical scenarios were established by making the leading vehicle decelerate from 80 km/h to 30 km/h at 3 m/s^2^ or change lane before the obstacle. Through changing the value of THW, three levels of critical scenarios (least critical, medium critical, and most critical) were set to investigate drivers’ takeover performance in critical brake situations [17]. With a leading vehicle running ahead, drivers of the ego vehicle could experience takeover scenarios with varying objective criticality by changing the THW and deceleration rate of the leading vehicle. In this context, the comparison of drivers’ takeover performance between critical and non-critical scenarios could be conducted in the car-following situation. Although several previous studies have examined the takeover performance in different critical scenarios in the car following situation, the non-critical scenario was not included. Moreover, as one of the important environmental factors affecting road safety, visibility significantly affects the driver’s behaviors [18,19]. Previous research has confirmed that reduced visibility in foggy conditions could increase the risk of collisions in the car-following situation [18]. Therefore, it is necessary to take the visibility into consideration when investigating the takeover risk from automation system in the car following scenarios. Several studies have examined the effects of visibility (in fog condition) on the takeover performance [20,21,22]. However, the findings in these studies are not in agreeance with each other, and more relevant studies are needed.

Therefore, this study aimed at filling the research gaps mentioned above with the following aspects: (i) investigating the effects of low visibility and time headway on the takeover performance in the L3 automation stage by conducting a driving simulator experiment; (ii) both non-critical and critical scenarios are taken into consideration by changing the acceleration value of the leading vehicle. The takeover time including the takeover reaction time and takeover control time was examined and analyzed by ANOVA (Analysis of Variance). A binary logit model was estimated to evaluate the takeover risk. The results presented in this study are expected to facilitate the overall understanding of the effects of visibility and time headway on the takeover risk and make a contribution to the improvement of takeover safety in conditionally automated driving.

The rest of this paper is organized as follows. Section 2 presents the driving simulator experiment, data collection and analysis methods. The results a are given in Section 3, followed by discussions in Section 4. Finally, Section 5 concludes the paper.

## 2. Methodology

### 2.1. Participants

A total of 35 participants were recruited among students and professional drivers at Tongji University for the driving simulator experiment. Each of them held a valid full driver’s license for at least two years. Moreover, each participant was in good health and had driving experience in foggy conditions. A questionnaire containing information about the driving experiment and some cautions was completed by each participant. Before conducting the experiment, informed consent was obtained from all subjects. Since 5 participants failed to complete the experiment as required due to driving simulator sickness, data from them were excluded from the analysis. The remaining 30 participants (12 females and 18 males) were aged from 21 to 51 years old (M = 30.2, SD = 8.6), and the average driving experience of these subjects was 6.6 years (SD = 4.7). Participants who met the following requirements were labeled as experienced drivers: (a) having more than three years of driving experience, (b) having an average annual driving mileage over 10,000 km during the last 3 years, (c) and not being involved in any accidents or crashes. Based on these three criteria, 14 participants were classified as experienced drivers.

### 2.2. Apparatus

**Figure 1:**
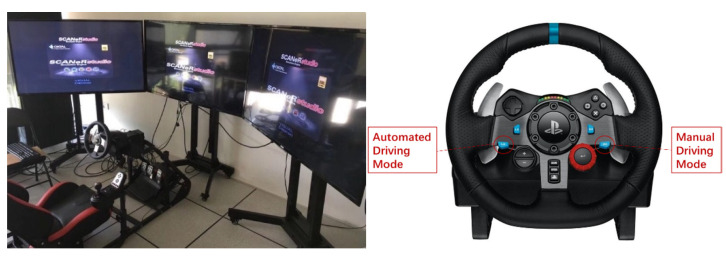
The driving simulator equipment used in this study.

The experiment was conducted based on an integrated driving simulation platform as shown in Figure 1, which was used in the previous study by Li et al. [2] to perform the driving simulator experiment. The driving simulation platform is mainly composed of the Logitech G29 driving simulation suite and SCANeR Studio, commercial software developed by the Oktal Co., Ltd. (Toulouse, France)). The driving simulation suite consists of pedals, a seat, and a steering wheel with dual motor force feedback technology. Two buttons on the steering wheel are set to transfer the mode from automated driving to manual driving or vice versa. The projection system comprises 3 TV screens, providing a frontal field of view about 135° × 40° (horizontal × vertical) from the driver’s perspective. The views of the rearview mirror and two side mirrors are also projected on the screens. The instrument panel is displayed at the bottom of the middle screen, showing the driver important information about the status of the vehicle. The sound system is used to output the takeover prompt as well as reproduce the environmental and vehicular noise.

### 2.3. Experimental Design

**Figure 2:**
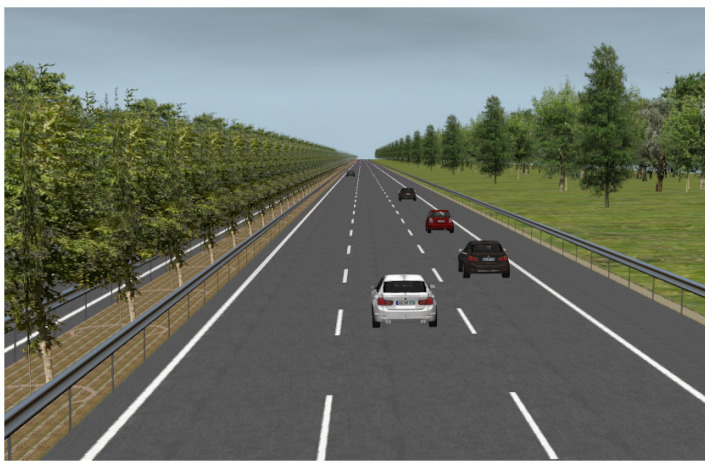
One of the driving scenarios displayed on the screen.

The objective of this study is to investigate the takeover performance. The experimental scenarios are based on a three-lane straight highway with a continuous emergency lane on the right side (Figure 2). This kind of highway scenario has been used in many previous studies [2,12,16], mainly for two reasons: (i) highways have a closed road environment and less interference compared with complex urban roads or rural roads, which are more suitable for the application of automated vehicles; (ii) the two-way six-lane highway or the one-way three-lane highway is very common, and a comparison between the driving simulation test and on-road test could be conducted in the future. For the automated conditions, the ego vehicle maintained a speed of 80 km/h in the right lane and followed a leading vehicle with the same speed. The automated driving of the ego vehicle and the transition between automated driving mode and manual driving mode were realized by the driving simulation system of SCANeR Studio.

**Figure 3:**
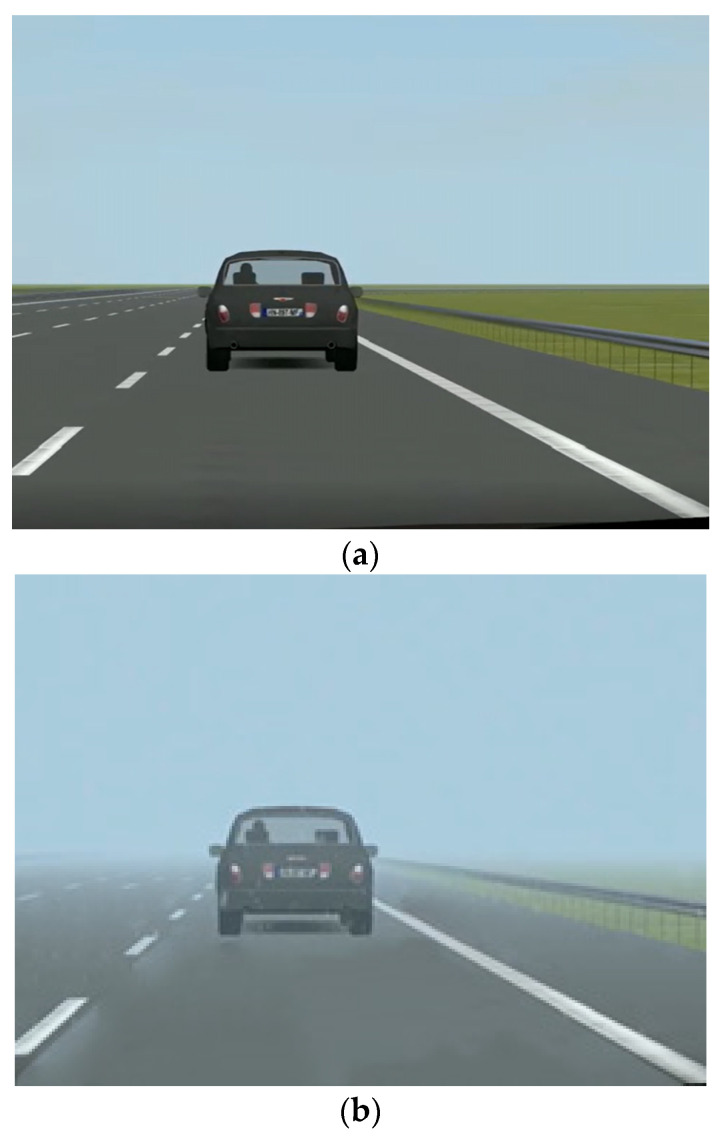
The visibility scene used in this study: (**a**) clear weather with visibility >1000 m and (**b**) fog condition with the visibility of 140 m.

A 2 × 3 × 3 within-subject experimental design was employed with three independent factors (level of visibility, following distance, and emergency level). For the visibility, as shown in Figure 3, the visibility distances of clear weather and light-fog conditions were set at >1000 m and 140 m, respectively [18]. The time headway (THW) between the ego vehicle and the leading vehicle was used to represent the following distance. Three levels of following distance (THW = 2 s, THW = 3 s, THW = 4 s) were employed in the driving scenarios. As the same time of the initial of takeover prompts, the acceleration of the leading vehicle (ALV) was applied to produce three emergency levels of takeover scenarios. ALV = 0 m/s^2^ means a non-critical scenario, and ALV = −2 m/s^2^ or −4 m/s^2^ means a critical scenario. Therefore, a total of 18 scenarios (see Table 1) were included in the driving simulator experiment. 

### 2.4. Experiment Procedure

Firstly, participants were required to complete a questionnaire with items about personal characteristics, such as gender, age, driving experience and so on. Then, participants were given instructions on the driving simulation system, takeover requests, as well as the experiment procedures. Next, each participant was required to perform a 5-minute practice drive to become familiar with the driving simulator and the takeover process. Since these participants have the experiences of driving simulator experiments using the same platform, the practice driving of 5 min is suitable in this study. 

**Figure 4:**
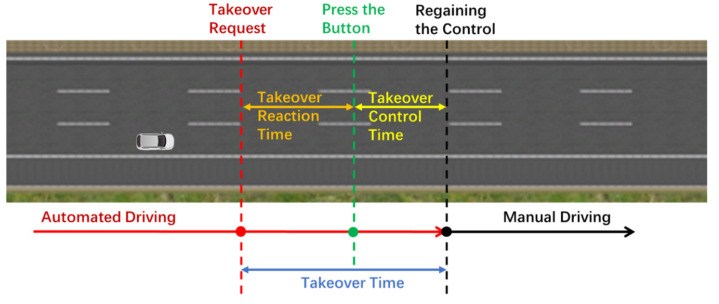
The takeover procedure and defines of takeover-related time.

After all these preparations, the formal experiment including 18 driving scenarios began. For each driving scenario, the participant needed to complete the whole process of “manual driving–automated driving–manual driving”. Firstly, the participant should start the ego car and speed it up to around 80 km/h. Then, the participant could transfer the control to the automation system by pressing the button of “Automated Driving Mode” on the steering wheel. Once entering the automated driving mode, the ego vehicle’s speed would be adjusted exactly to 80 km/h and the time headway would be smoothly adjusted to a certain value (2 s, 3 s, or 4 s) by the driving simulation system. During the automated driving, participants were required to watch videos on a tablet computer. Once they heard the takeover requests of “Please Takeover”, they were required to press the button “Manual Driving Mode” and takeover the ego vehicle in a timely fashion, as shown in Figure 4. Participants could keep following the leading vehicle or choose to change lanes according to their willingness. To eliminate the effect of experimental sequence, participants were assigned to 18 driving scenarios in a random order.

### 2.5. Data Collection, Measures, and Analysis

Data related to the drivers’ operational behaviors and the vehicle’s running state were collected with the frequency of 20 Hz through the driving simulator system. A document in the form of CSV was output for each participant after the experiment of one driving scenario. In total, 540 CSV files were obtained at the end of the driving simulator experiment in this study. As for the ego vehicle, the speed, acceleration, and position data were collected. As for the takeover process, the time of pressing the button, the time of pushing the gas pedal or the brake pedal, and the time of steering were output.

Based on the data obtained from the driving simulation system, some measures of interest were extracted or calculated. As shown in Figure 4, takeover time refers to the time between the takeover request and the driver regaining the control of the vehicle. Mainly three kinds of takeover responses (pushing the brake pedal, pushing the gas pedal, and manipulating the steering wheel) were conducted by participants to regain the vehicle control. The criteria of a driver regaining the vehicle control are set as follows: (i) pushing the brake or gas pedal over 10% of the range; (ii) the steering wheel angle changes over 2 degrees. According to the criteria above, the time when a driver regains the vehicle control could be extracted and the first response to regaining the vehicle control was the takeover response of the driver. Then, the takeover time could be obtained with the time of takeover request and the time of regaining the vehicle control. In this study, the takeover time was divided into two parts: (i) takeover reaction time (TRT) and (ii) takeover control time (TCT). As displayed in Figure 4, TRT is the time from takeover request to pressing the button of automated driving mode, and TCT refers to the time between pressing the button and regaining the vehicle control.

The minimum time-to-collision (Min TTC) [12,21] that occurred within the takeover process was derived in order to measure the takeover risk. The Min TTC is an effective measure to assess the risk of potential collisions. In this study, the Min TTC refers to the minimum time required for the ego vehicle to collide with the leading vehicle during the whole takeover process, which is calculated as follows (Δd is the distance between the ego vehicle and the leading vehicle; t is the time; L is the length of the leading vehicle; V2 is the speed of the ego vehicle and *V*1 is the speed of the leading vehicle):

(1) $$Min\;TTc=\min(\frac{\Delta d(t)-L}{V2(t)-V1(t)})$$

ANOVA (Analysis of Variance) was carried out on continuous variables, and a binary logit model [13] was applied to examine the risk of takeover process. In this study, the Min TTC threshold of 1.5 s was used to define the takeover risk level (High Risk with the Min TTC < 1.5 s and Low Risk with the Min TTC ≥ 1.5 s). Specifically, the dependent variable Y representing the takeover risk level is a binary variable that can take on two values:

(2) $$Y=\{\begin{array}{c} 1,\;denoting\;High\;Risk\;with\;the\;Min\;TTC<1.5\;s\\ 0,\;denoting\;Low\;Risk\;with\;the\;Min\;TTC\;\geq 1.5\;s \end{array}$$

Suppose the conditional probability of the occurrence of the outcome is modelled as a logistic distribution:

(3) $$\;P\left(Y=1\right)=\pi =\frac{\exp\left(\alpha +\beta_{i}x_{i}\right)}{1+\exp\left(\alpha +\beta_{i}x_{i}\right)}$$

(4) $$P\left(Y=0\right)=1-\pi =\frac{1}{1+\exp\left(\alpha +\beta_{i}x_{i}\right)}$$

Then, the odds of High Risk (y=1) happening is π/(1−π), and the binary logit model has the form

(5) $$logit\left(Y\right)=In\left(\frac{\pi }{1-\pi }\right)=\alpha +\beta_{i}x_{i}$$

where π is the conditional probability of the High Risk occurrence; $x_{i}$ denotes independent variables, which can be categorical or continuous; α is the intercept and $\beta_{i}$ is the regression coefficient associated with each independent variable. The values of α and $\beta_{i}$ are typically estimated by the maximum likelihood (ML) method, which is designed to maximize the likelihood of reproducing the data given the parameter estimates [23].

The value of $\beta_{i}$ determines the direction of the relationship between $x_{i}$ and the logit of Y. $\beta_{i}>0$ means larger $x_{i}$ values are associated with larger logit of Y, and conversely, $\beta_{i}<0$ means larger $x_{i}$ values are associated with smaller logit of Y. Generally, the odds ratio OR = exp($\beta_{i}$) is applied to interpret the estimated results for both categorical and continuous independent variables. OR > 1 (or OR < 1) indicates the odds of outcome increases (or decreases) when the corresponding independent variable increases by one unit, keeping other variables constant.

## 3. Results

### 3.1. Takeover Time

**Figure 5:**
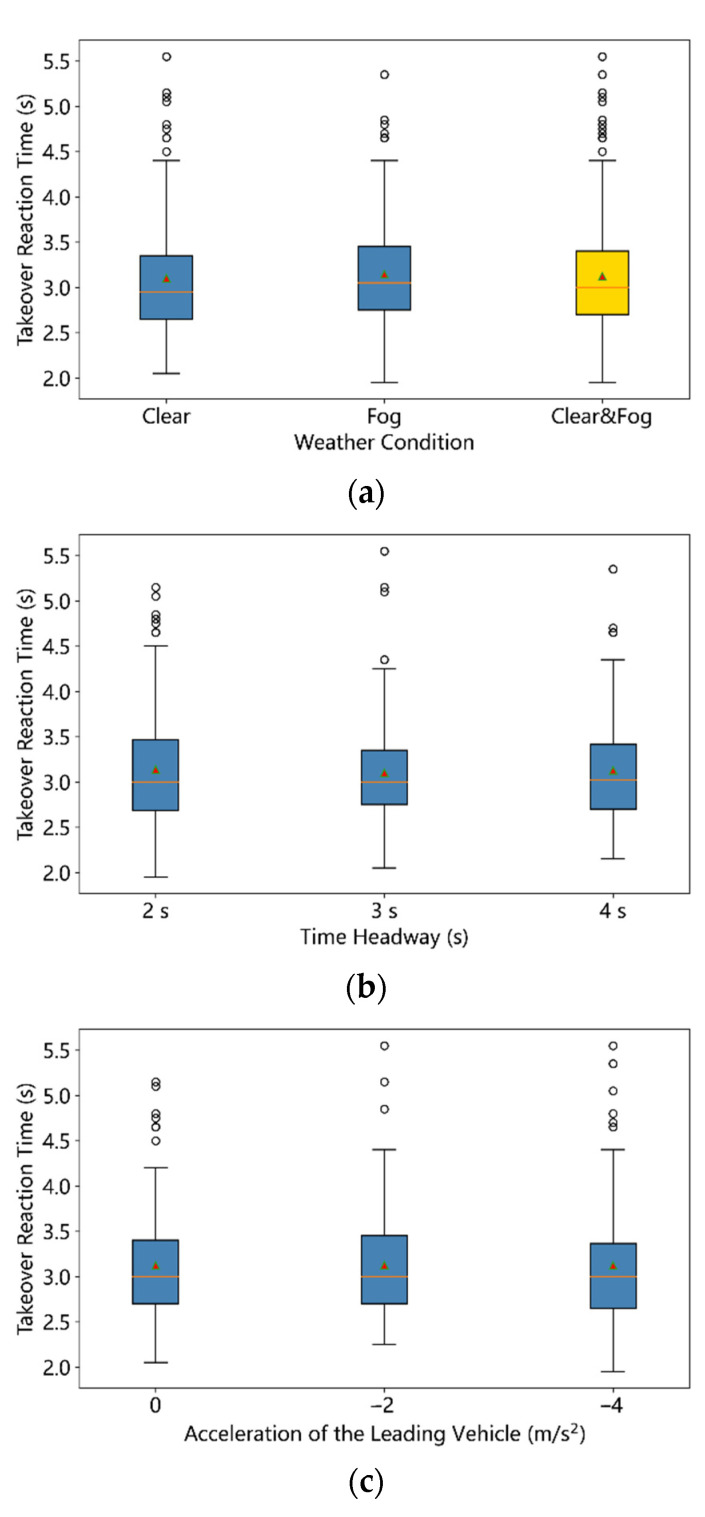
Boxplot of takeover reaction time of all 540 samples: (**a**) Weather Condition, (**b**) Time Headway, (**c**) Acceleration of the Leading Vehicle (the red triangle symbol denotes the mean value).

As mentioned above, the takeover time could be divided into the takeover reaction time (TRT) and the takeover control time (TCT). The statistical summary of the TRT was listed in Table 2. Taking all the samples into consideration, the TRT values ranged from 1.95 s to 5.55 s, with the mean value of 3.12 s (Std = 0.62 s). As can be seen from Figure 5, three factors (VD, THW, and ALV) seem to exert little influence of the TRT. A three-way ANOVA analysis was conducted to examine the influence. Results shown in Table 3 indicate that all three factors have no significant effects on the TRT under the confidence level of 95%. Moreover, no reliable interaction between any two of these factors exists according to the results.

**Figure 6:**
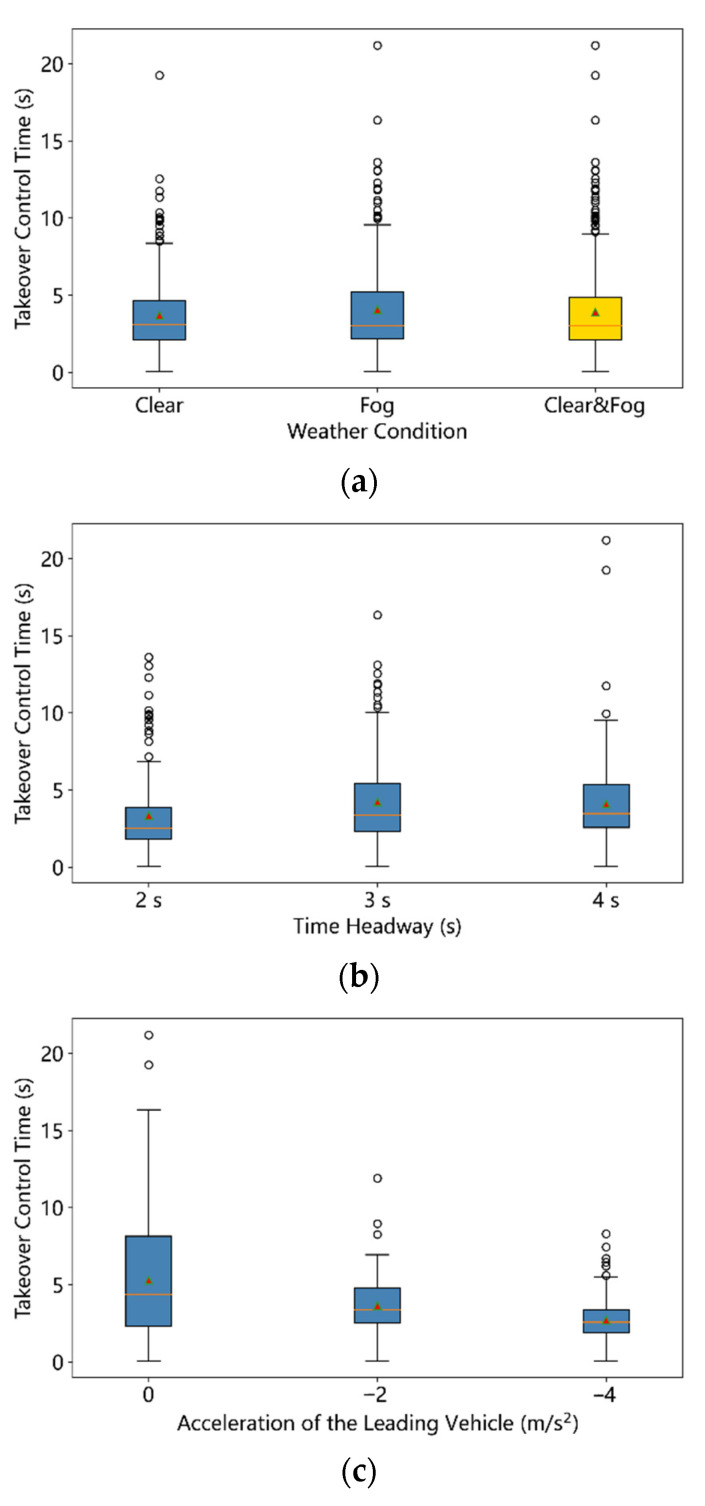
Boxplot of takeover control time of all 540 samples: (**a**) Weather Condition, (**b**) Time Headway, (**c**) Acceleration of the Leading Vehicle (the red triangle symbol denotes the mean value).

The takeover control time (TCT) refers to the time between pressing the button and regaining the control of the vehicle. Table 4 displays the statistical summary of TCT. The TCT values of all the 540 samples ranged from 0.05 s to 21.20 s, with a mean value of 3.89 s and standard deviation value of 2.81 s. Since the data collection frequency was 20 Hz, the 0.05 s means that the driver regained the vehicle control almost at the same time of pressing the button of takeover mode. Table 5 presents the ANOVA results of three factors on the takeover control time. The three-way ANOVA results indicate that the VD has no significant effects on the takeover control time, while the THW (F = 6.01, *p* = 0.0026 < 0.01) and the ALV (F = 46.08, *p* < 0.001) could affect the takeover control time significantly. No significant interaction effect between THW and ALV was found (F = 1.13, *p* = 0.341). The effects of time headway on the takeover control time could be seen in the Figure 6b, which shows the takeover control time under the THW = 2 s condition is smaller significantly than that of THW = 3 s and THW = 4 s. However, the takeover control time of THW = 3 s has no significant difference with that of THW = 4 s from the result of multiple comparison. Figure 6c presents the effects of ALV on the takeover control time, which indicates that the takeover control time becomes smaller as the ALV decreases. Compared with the driving scenarios of ALV = 0, scenarios with ALV = −2 m/s^2^ or −4 m/s^2^ are in emergency. The time budget for drivers to avoid a potential accident is smaller as the ALV changes from −2 m/s^2^ to −4 m/s^2^, so drivers need to take over the ego vehicle more quickly in the driving scenarios of ALV = −4 m/s^2^. More specifically, the mean value of takeover control time decreases from 3.65 s (SD = 1.94 s) to 2.73 s (SD = 1.34 s) as the ALV changes from −2 m/s^2^ to −4 m/s^2^, much smaller than mean value 5.29 s (SD = 3.85 s) of non-critical scenarios (ALV = 0).

### 3.2. Takeover Responses

**Figure 7:**
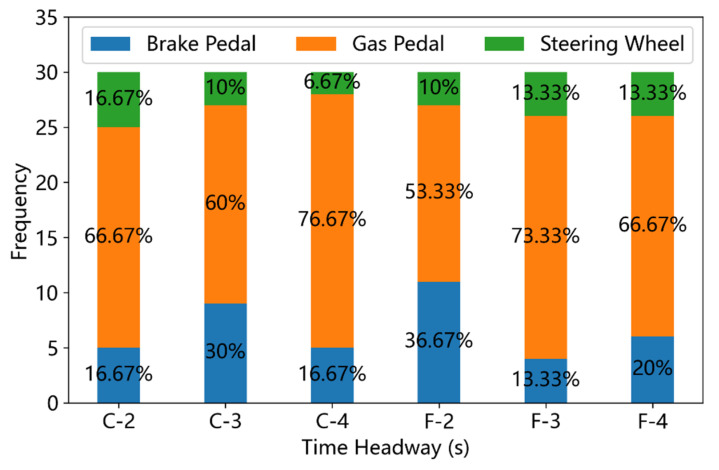
Frequency of different takeover responses under non-critical driving scenarios (C means ‘clear weather’ and F denotes ‘fog condition’).

**Figure 8:**
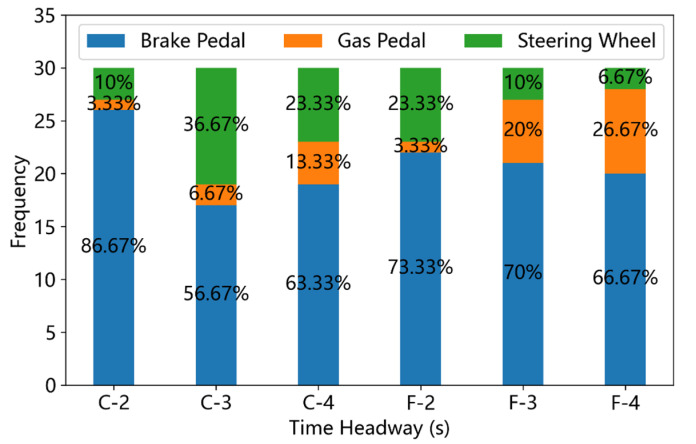
Frequency of different takeover responses under sub-critical (ALV = −2 m/s^2^) driving scenarios (C means ‘clear weather’ and F denotes ‘fog condition’).

**Figure 9:**
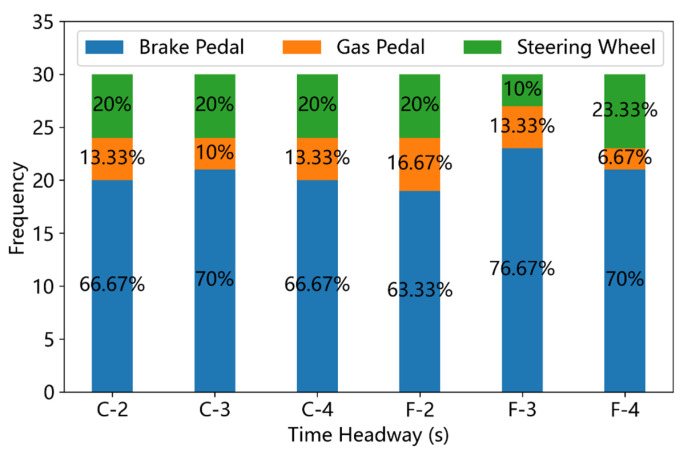
Frequency of different takeover responses under most-critical (ALV = −4 m/s^2^) driving scenarios (C means ‘clear weather’ and F denotes ‘fog condition’).

Three takeover responses (pushing the brake pedal, pushing the gas pedal, and manipulating the steering wheel) were observed during the driving simulator experiments in this study. The frequencies and ratios of these takeover responses under different driving conditions were displayed in Figure 7, Figure 8 and Figure 9. In non-critical scenarios (ALV = 0), over 50% takeover responses are sped up, as shown in Figure 7. In critical scenarios (Figure 8 and Figure 9), pushing the brake accounts for over 55% of the takeover responses and less than 30% takeover responses are sped up. The Pearson chi-square test indicates that the ALV did possess significant effects on the choice of takeover responses ($\chi^{2}=164.26,\;p<0.001)$. 

In the non-critical scenarios, the leading vehicle keeps the speed of 80 km/h all the time, so the distance between the ego vehicle and the leading vehicle would not decrease, even if the participant did not take any brake operation. In this circumstance, most drivers chose to push the gas pedal to accelerate the ego vehicle. Moreover, as can be seen in Figure 7, the maximum rate (36.67%) of pushing the brake pedal occurred in the condition of Fog weather with the THW = 2 s, and the maximum rate (76.67%) of pushing the gas pedal occurred in the condition of Clear weather with the THW = 4 s. However, the Pearson chi-square test shows that the VD ($\chi^{2}=0.22,\;p=0.894)$ and THW ($\chi^{2}=1.86,\;p=0.762)$ have no significant effects on the type of takeover responses in non-critical scenarios.

For the sake of description, driving scenarios with ALV = −2 m/s^2^ are called sub-critical scenarios, and driving scenarios with ALV = −4 m/s^2^ are named most-critical scenarios. Figure 8 and Figure 9 display the frequency and ratio of different takeover responses under sub-critical and most-critical driving scenarios, respectively. Compared with the bar plot in Figure 7, it is obvious that the braking is the dominant takeover response for participants in critical driving scenarios. In total, 86.7% participants chose to push the brake pedal as the takeover response in the clear weather with the THW = 2 s. Moreover, the number of participants who chose to manipulate the steering wheel as the takeover response increases from 3 to 11 when the ALV changes from 0 to −2 m/s^2^ in clear weather with the THW = 3 s. It should be noted that in the sub-critical driving scenarios, more participants took the speed-up or pushing the gas pedal as their takeover response under the fog condition with THW = 3 s and THW = 4 s than that of clear weather. This phenomenon could result from the lower recognition of distance in the fog condition, which made the drivers misestimate the following distance. In the sub-critical scenarios, the Pearson chi-square test result indicates that the effects of THW ($\chi^{2}=9.64,\;p=0.047)$ on the takeover responses is significant while the effects of VD ($\chi^{2}=5.37,\;p=0.068)$ on the takeover responses is insignificant. In the most-critical scenarios, the ratios of pushing the brake pedal are all over 60%, and the Pearson chi-square test result indicates that both the VD ($\chi^{2}=0.20,\;p=0.905)$ and THW ($\chi^{2}=0.99,\;p=0.911)$ exert insignificant influence on the choice of takeover responses.

### 3.3. Takeover Risk Analysis

The Min TTC between the ego vehicle and the leading vehicle during the takeover process was employed to evaluate the risk of takeover in this study. Since in the non-critical scenarios, as mentioned before, the speed difference between the ego vehicle and the leading vehicle is near zero when pressing the button of taking over the vehicle control, the Min TTC values are too large to cause a rear-end collision or any other possible accidents. Thus, 180 samples of the non-critical scenarios were excluded from the risk analysis below. For the critical scenarios, 360 Min TTC values during the takeover process were calculated and the summary was listed in Table 6. Min TTC = 0 means a rear-end collision occurred during the takeover process, and 12 rear-end collisions occurred totally in the critical scenarios in this study. Based on the previous studies about TTC, 1.5 s was selected as the threshold value to define the risk levels during takeover process. As represented in the Section 2.5, the Min TTC less than 1.5 s was defined as High Risk (Y = 1) and the Min TTC larger than 1.5 s was defined as Low Risk (Y = 0). 

A binary logit model was applied to examine the effects of explanatory variables on the takeover risk levels. The risk level was considered as the dependent variable and Low Risk was set as the base level. Therefore, all the estimated coefficients for the selected explanatory variables indicate the effect of the variables on the High Risk level compared with the base level. Table 7 presents the descriptive statistics of these potential explanatory variables in which the mean, standard deviation, minimum and maximum of these variables are given. Since data of non-critical driving scenarios were not included in the takeover risk analysis, 360 samples were used to conduct the binary logit model. 

Table 8 presents the results of the binary logit model for takeover risk levels in the critical driving scenarios in this study. It should be noted that statistically insignificant variables are excluded from the model. The variance inflation factor (VIF) was employed to test the multicollinearity of the model. Since each explanatory variable showed a VIF < 2, there is no multicollinearity issue in the logit model. The result of Hosmer–Lemeshow (H-L) test ($\chi^{2}\left(8\right)=6.397,\;p=0.603>0.05$) suggested that the model was fit to the data well. According to the *p*-value, the results show that compared with Low Risk level, variables THW, ALV, TR, and TCT affected the High Risk level significantly. As for the THW, compared with THW2, the odds of High Risk occurrence decrease from 1.0 to 0.549 when the THW = 3 s. Similarly, if the THW = 4 s, the odds of High Risk occurrence would decrease from 1.0 to 0.192 if the THW changed from 2 s to 4 s. As for the ALV, the odds of High Risk occurrence under the condition of ALV = −4 m/s^2^ was 2.784 times greater than the odds under the condition of ALV = −2 m/s^2^. The coefficient of the TR was negative, which means that the odds of High Risk occurrence when pushing the brake pedal would decrease from 1.0 to 0.221 compared with other takeover responses. This amount of decrease in odds revealed the importance of pushing the brake pedal during the takeover process in critical scenarios in this study. As for the TCT, which is a continuous variable, the odds ratio indicates the odds of High Risk would increase from 1.0 to 1.241 as the TCT increases by 1 s.

## 4. Discussion and Limitations

This study aimed to investigate the influence of the visibility distance (VD) and the time headway (THW) on the takeover risk under the conditionally automated driving situation. Both non-critical and critical driving scenarios were provided to the drivers involving the driving simulator experiment by changing the value setting of the acceleration of the leading vehicle (ALV).

As for the takeover time, both takeover reaction time (TRT) and takeover control time (TCT) were analyzed. No significant differences in TRT among different driving scenarios were observed in this study, which suggests that these participants tended to press the button of manual driving mode at their own pace once they received takeover requests. The urgency of the scenario imposed limited influence on the TRT. This is consistent with previous studies reviewed in Eriksson and Stanton [14], which indicated that the values of TRT stay fairly consistent in most control transitions. This could be explained by participants’ understanding of the necessary of takeover during conditionally automated driving. Before conducting the experiment, participants were informed about the takeover process and the need to avoid collisions. Thus, most of the participants tended to press the button even though they did not confirm the urgency of the situations ahead. Moreover, before hearing the takeover requests, all the participants were performing the same non-driving related task in this study, so the effects of NDRTs on the TRT could be neglected. 

The takeover control time (TCT) was influenced by THW and ALV significantly in this study, which suggested that the urgency of the scenario could affect the TCT of these participants. This indicates that for most of the drivers, they would confirm the risk of the situation ahead before making an adequate decision and response to regain the vehicle control [24]. As the ALV decreases, which means the driving scenario becomes more critical, the mean value and the maximum of TCT decrease significantly. This is consistent with the finding from previous research [25] that drivers would take over faster when given a shorter time budget compared a longer time budget. Although a significant decrease in TCT when THW = 2 s was found compared with larger THW values, the difference in TCT between THW = 3 s and THW = 4 s was not significant. This could be explained in terms of the fact that the THW over 3 s means a safe car-following situation even for conservative drivers [26,27]. 

The VD levels in this study did not bring about a significant influence on the takeover time or takeover responses, as well as the takeover risk. This could be induced by the fact that the visibility distance (140 m) in the fog condition was larger than the car following distance in all the driving scenarios. Although the fog would impair the clarity of the front view of drivers, they could still spot the leading vehicle and recognize if the leading vehicle was braking or not according to the tail lamp. Therefore, the effects of VD on the drivers’ TCT and takeover responses are limited in this study.

The result of the binary logit model highlights the significant difference of takeover risk between the sub-critical (ALV = −4 m/s^2^) and most-critical (ALV = −4 m/s^2^) driving scenarios. Although the TCT decreases as the ALV decreases, the takeover risk increases significantly due to the decrease in the time-budget in most-critical scenarios compared with the sub-critical scenarios. The timely brake by the drivers is very important to reduce the takeover risk, as revealed by the result of the binary logit model. Taking the TCT and the takeover response into consideration, decreasing the TCT and taking the appropriate takeover response would reduce the takeover risk significantly. If the urgency of the driving scenario or the time budget for takeover is constant, improving the drivers’ ability to realize the risk of the situation quickly and take correct responses would benefit the safety of the takeover process in the conditionally automated driving. Therefore, it is suggested that the automation system should provide the driver with the urgency of the situation ahead and the tips for takeover responses by audio prompts or the head-up display.

There are several limitations in this study, which should be considered when interpreting the results. First, the VD values are larger than the car-following distance in all the driving scenarios, which could not reveal the effects of heavier fog conditions on the takeover risk. More VD values corresponding to different kinds of fog conditions [18] are needed in future studies. Second, participants in the driving simulator experiment were requested to conduct the same non-driving-related task, which would not be the case in real driving processes. The effects of NDRT should be included in future studies. Finally, in order to set up a controlled experiment, the speed of the leading vehicle is the same under different VD conditions. It should be noted that a lower vehicle speed is more appropriate in a heavier fog condition.

## 5. Conclusions

To examine the effects of the visibility distance (VD) and the time headway (THW) on the takeover performance in conditionally automated driving, a driving simulator experiment was conducted. Both non-critical and critical scenarios were taken into consideration by adjusting the acceleration of the leading vehicle (ALV). A 2 × 3 × 3 within-subject experimental design was employed with three independent factors (VD, THW, and ALV), which results in 18 driving scenarios in the driving simulator experiment. The takeover reaction time (TRT), takeover control time (TCT) and takeover responses (TR) were analyzed based on the data from 30 participants. Moreover, the binary logit model for takeover risk was estimated, in which the Min TTC was employed to set the risk levels. The conclusions are summarized as follows:

(i) The mean value of the takeover reaction time is 3.12 s with the standard deviation of 0.62 s. All three factors (VD, THW and ALV) have no significant effects on the takeover reaction time. The VD has no significant effects on the takeover control time, while the THW and the ALV could affect the takeover control time significantly.

(ii) Most of the participants would push the gas pedal to accelerate the ego vehicle as the takeover response under non-critical scenarios, while braking is the dominant takeover response for participants in critical driving scenarios.

(iii) Decreasing the TCT and taking the appropriate takeover response would reduce the takeover risk significantly. Therefore, it is suggested that the automation system should provide the driver with the urgency of the situation ahead and the tips for takeover responses by audio prompts or the head-up display.

In summary, this study provides some valuable information for better understanding the effects of visibility and time headway on the takeover performance and is expected to be helpful to improve the safety of takeover process in conditionally automated driving. However, just two visibility levels were considered in this study. More levels of visibility should be designed in the driving scenarios in future studies.

## Figures and Tables

**Table 1:** Labels and three independent factors of 18 driving scenarios.

Label of the Scenario	Visibility Distance (VD)	Time Headway (THW)	Acceleration of the Leading Vehicle (ALV)
	(>1000 m, 140 m)	(2 s, 3 s, 4 s)	(0 m/s^2^, −2 m/s^2^, −4 m/s^2^)
C-2-0	>1000 m	2 s	0 m/s^2^
C-2-2	>1000 m	2 s	−2 m/s^2^
C-2-4	>1000 m	2 s	−4 m/s^2^
C-3-0	>1000 m	3 s	0 m/s^2^
C-3-2	>1000 m	3 s	−2 m/s^2^
C-3-4	>1000 m	3 s	−4 m/s^2^
C-4-0	>1000 m	4 s	0 m/s^2^
C-4-2	>1000 m	4 s	−2 m/s^2^
C-4-4	>1000 m	4 s	−4 m/s^2^
F-2-0	140 m	2 s	0 m/s^2^
F-2-2	140 m	2 s	−2 m/s^2^
F-2-4	140 m	2 s	−4 m/s^2^
F-3-0	140 m	3 s	0 m/s^2^
F-3-2	140 m	3 s	−2 m/s^2^
F-3-4	140 m	3 s	−4 m/s^2^
F-4-0	140 m	4 s	0 m/s^2^
F-4-2	140 m	4 s	−2 m/s^2^
F-4-4	140 m	4 s	−4 m/s^2^

Note. ‘C’ denotes the clear weather with the visibility distance >1000 m; ‘F’ denotes the light fog condition with the visibility distance of 140 m.

**Table 2:** Summary of the takeover reaction time.

Conditions		Mean (s)	Std (s)	Min (s)	Max (s)
**VD**	Clear	3.10	0.63	2.05	5.55
	Fog	3.15	0.61	1.95	5.35
**THW**	2	3.14	0.68	1.95	5.15
	3	3.10	0.60	2.05	5.55
	4	3.13	0.59	2.15	5.35
**ALV**	0	3.12	0.63	2.05	5.15
	−2	3.13	0.58	2.25	5.55
	−4	3.12	0.66	1.95	5.55
**All 540 Samples**		3.12	0.62	1.95	5.55

**Table 3:** Three-way ANOVA results on takeover reaction time.

Source	Sum Sq.	d.f.	Mean Sq.	F	Prob > F
**VD**	0.269	1	0.269	0.68	0.4085
**THW**	0.136	2	0.068	0.17	0.8407
**ALV**	0.003	2	0.001	0	0.9963
**VD × THW**	0.85	2	0.425	1.08	0.3398
**VD × ALV**	0.512	2	0.256	0.65	0.5212
**THW × ALV**	0.38	4	0.095	0.24	0.9145
**Error**	206.595	526	0.393		
**Total**	208.745	539			

**Table 4:** Summary of the takeover control time.

Conditions		Mean (s)	Std (s)	Min (s)	Max (s)
**VD**	Fog	3.71	2.52	0.05	19.25
	Clear	4.07	3.08	0.05	21.20
**THW**	2	3.35	2.69	0.05	13.60
	3	4.23	2.95	0.05	16.35
	4	4.09	2.74	0.05	21.20
**ALV**	0	5.29	3.86	0.05	21.20
	−2	3.65	1.94	0.05	11.90
	−4	2.72	1.34	0.05	8.30
**All 540 Samples**		3.89	2.81	0.05	21.20

**Table 5:** Three-way ANOVA results on takeover control time.

Source	Sum Sq.	d.f.	Mean Sq.	F	Prob > F
**VD**	17.24	1	17.244	2.60	0.1072
**THW**	79.54	2	39.77	6.01	0.0026 **
**ALV**	610.22	2	305.11	46.08	<0.001 ***
**VD × THW**	21.59	2	10.797	1.63	0.1968
**VD × ALV**	29.65	2	14.823	2.24	0.1076
**THW × ALV**	29.95	4	7.488	1.13	0.341
**Error**	3482.66	526	6.621		
**Total**	4270.86	539			

Note. **, and *** denote significance at the 99%, and 99.9% confidence levels, respectively.

**Table 6:** Statistical summary of Min TTC in the critical scenarios.

Conditions	Mean (s)	Std (s)	Min (s)	Median (s)	Max (s)
**All 360 Samples**	1.57	1.13	0	1.44	10.36

**Table 7:** Descriptive statistics of the explanatory variables.

Name	Variable	Coding	Mean	Std	Max	Min
**Visibility Distance**	**VD**	1 (fog); 0 (clear)	0.500	0.500	1	0
**Gender**	**Gd**	1 (female); 0 (male)	0.400	0.491	1	0
**Driving Experience**	**DE**	1 (novice driver); 0 (experienced driver)	0.467	0.500	1	0
**Time Headway (THW)**						
THW = 2 s	**THW2**	1 (THW = 2 s); 0 (others)	0.333	0.472	1	0
THW = 3 s	**THW3**	1 (THW = 3 s); 0 (others)	0.333	0.472	1	0
THW = 4 s	**THW4**	1 (THW = 4 s); 0 (others)	0.333	0.472	1	0
**Acceleration of Leading Vehicle (ALV)**	**ALV**	1 (ALV = −4 m/s^2^); 0 (ALV = −2 m/s^2^)	0.500	0.500	1	0
**Takeover Response**	**TR**	1 (braking); 0 (others)	0.692	0.462	1	0
**Takeover Reaction Time**	**TRT**	Continuous variable	3.126	0.622	5.55	1.95
**Takeover Control Time**	**TCT**	Continuous variable	3.185	1.731	11.90	0.050

**Table 8:** Results of the binary logit model for takeover risk.

	β	Std. Error	$\mathrm{Wald}’s\;\chi^{2}$	df	*p*-Value	Exp(β)	95% Confidence Interval	
							Lower	Upper
**Intercept**	−0.712	0.386	3.406	1	0.065			
**THW3**	−0.600	0.296	4.111	1	0.043 *	0.549	0.307	0.980
**THW4**	−1.652	0.315	27.472	1	0.000 ***	0.192	0.103	0.356
**ALV**	1.024	0.250	16.814	1	0.000 ***	2.784	1.707	4.542
**TR**	−1.508	0.276	29.771	1	0.000 ***	0.221	0.129	0.381
**TCT**	0.216	0.079	7.369	1	0.007 **	1.241	1.062	1.450
***Model Statistics***								
Number of Observations		360						
Hosmer–Lemeshow			$6.397\;\left(\chi^{2}\right)$	8	0.603			

Note. *, **, and *** denote significance at the 95%, 99%, and 99.9% confidence levels, respectively.

## Data Availability

The data that support the findings of this study are available from the corresponding author, upon reasonable request.

## References

[1] Choi D., Sato T., Ando T., Abe T., Akamatsu M., Kitazaki S. (2020). Effects of cognitive and visual loads on driving performance after take-over request (TOR) in automated driving. Appl. Ergon..

[2] Li C., Li X., Lv M., Chen F., Ma X., Zhang L. (2021). How Does Approaching a Lead Vehicle and Monitoring Request Affect Drivers’ Takeover Performance? A Simulated Driving Study with Functional MRI. Int. J. Environ. Res. Public Health.

[3] SAE International (2018). Taxonomy and Definitions for Terms Related to Driving Automation Systems for On-Road Motor Vehicles’ (J3016). https://www.sae.org/standards/content/j3016_201806/.

[4] Lin Q., Li S., Ma X., Lu G. (2020). Understanding take-over performance of high crash risk drivers during conditionally automated driving. Accid. Anal. Prev..

[5] Zhang H., Zhang Y., Xiao Y., Wu C. (2022). Analyzing the Influencing Factors and Workload Variation of Takeover Behavior in Semi-Autonomous Vehicles. Int. J. Environ. Res. Public Health.

[6] Soares S., Lobo A., Ferreira S., Cunha L., Couto A. (2021). Takeover performance evaluation using driving simulation: A systematic review and meta-analysis. Eur. Transp. Res. Rev..

[7] Pampel S.M., Large D.R., Burnett G., Matthias R., Thompson S., Skrypchuk L. (2019). Getting the driver back into the loop: The quality of manual vehicle control following long and short non-critical transfer-of-control requests: TI:NS. Theor. Issues Ergon. Sci..

[8] Xu C., Li P., Li Y., Merat N., Lu Z., Guo X. Takeover Performance and Workload under Varying Automation Levels, Time Budget and Road Curvature. Proceedings of the 2022 IEEE Asia-Pacific Conference on Image Processing, Electronics and Computers (IPEC).

[9] Naujoks F., Purucker C., Wiedemann K., Marberger C. (2019). Noncritical State Transitions During Conditionally Automated Driving on German Freeways: Effects of Non–Driving Related Tasks on Takeover Time and Takeover Quality. Hum. Factors J. Hum. Factors Ergon. Soc..

[10] Louw T., Merat N. (2017). Are you in the loop? Using gaze dispersion to understand driver visual attention during vehicle automation. Transp. Res. Part C Emerg. Technol..

[11] Gold C., Happee R., Bengler K. (2018). Modeling take-over performance in level 3 conditionally automated vehicles. Accid. Anal. Prev..

[12] Wu H., Wu C., Lyu N., Li J. (2022). Does a faster takeover necessarily mean it is better? A study on the influence of urgency and takeover-request lead time on takeover performance and safety. Accid. Anal. Prev..

[13] García A., Camacho-Torregrosa F.J., Padovani Baez P.V. (2020). Examining the effect of road horizontal alignment on the speed of semi-automated vehicles. Accid. Anal. Prev..

[14] Eriksson A., Stanton N.A. (2017). Takeover Time in Highly Automated Vehicles: Noncritical Transitions to and From Manual Control. Hum. Factors J. Hum. Factors Ergon. Soc..

[15] Huang Y., Yan X., Li X., Duan K., Rakotonirainy A., Gao Z. (2022). Improving car-following model to capture unobserved driver heterogeneity and following distance features in fog condition. Transp. A Transp. Sci..

[16] Lu G., Zhai J., Li P., Chen F., Liang L. (2021). Measuring drivers’ takeover performance in varying levels of automation: Considering the influence of cognitive secondary task. Transp. Res. Part F Traffic Psychol. Behav..

[17] Roche F., Thüring M., Trukenbrod A.K. (2020). What happens when drivers of automated vehicles take over control in critical brake situations?. Accid. Anal. Prev..

[18] Wang K., Zhang W., Feng Z., Yu H., Wang C. (2021). Reasonable driving speed limits based on recognition time in a dynamic low-visibility environment related to fog—A driving simulator study. Accid. Anal. Prev..

[19] Zhao X., Chen Y., Li H., Ma J., Li J. (2021). A study of the compliance level of connected vehicle warning information in a fog warning system based on a driving simulation. Transp. Res. Part F Traffic Psychol. Behav..

[20] Brandenburg S., Roche F. (2020). Behavioral changes to repeated takeovers in automated driving: The drivers’ ability to transfer knowledge and the effects of takeover request process. Transp. Res. Part F Traffic Psychol. Behav..

[21] Li S., Blythe P., Guo W., Namdeo A. (2018). Investigation of older driver’s takeover performance in highly automated vehicles in adverse weather conditions. IET Intell. Transp. Syst..

[22] Louw T., Markkula G., Boer E., Madigan R., Carsten O., Merat N. (2017). Coming back into the loop: Drivers’ perceptual-motor performance in critical events after automated driving. Accid. Anal. Prev..

[23] Peng C.-Y.J., Lee K.L., Ingersoll G.M. (2002). An Introduction to Logistic Regression Analysis and Reporting. J. Educ. Res..

[24] Weaver B.W., DeLucia P.R. (2020). A Systematic Review and Meta-Analysis of Takeover Performance During Conditionally Automated Driving. Hum. Factors J. Hum. Factors Ergon. Soc..

[25] Zhang B., de Winter J., Varotto S., Happee R., Martens M. (2019). Determinants of take-over time from automated driving: A meta-analysis of 129 studies. Transp. Res. Part F Traffic Psychol. Behav..

[26] Sun P., Wang X., Zhu M. (2021). Modeling Car-Following Behavior on Freeways Considering Driving Style. J. Transp. Eng. Part A Syst..

[27] Liu T., Fu R., Ma Y., Liu Z.-F., Cheng W.-D. (2020). Car-following Warning Rules Considering Driving Styles. Zhongguo Gonglu Xuebao/China J. Highw. Transp..

